# Measuring the effect of commuting on the performance of the Bayesian Aerosol Release Detector

**DOI:** 10.1186/1472-6947-9-S1-S7

**Published:** 2009-11-03

**Authors:** Aurel Cami, Garrick L Wallstrom, William R Hogan

**Affiliations:** 1 Children's Hospital Boston Informatics Program, Boston, MA, USA; 2 Department of Biomedical Informatics, University of Pittsburgh, Pittsburgh, PA, USA

## Abstract

**Background:**

Early detection of outdoor aerosol releases of anthrax is an important problem. The Bayesian Aerosol Release Detector (BARD) is a system for detecting releases of aerosolized anthrax and characterizing them in terms of location, time and quantity. Modelling a population's exposure to aerosolized anthrax poses a number of challenges. A major difficulty is to accurately estimate the exposure level--the number of inhaled anthrax spores--of each individual in the exposed region. Partly, this difficulty stems from the lack of fine-grained data about the population under surveillance. To cope with this challenge, nearly all anthrax biosurveillance systems, including BARD, ignore the mobility of the population and assume that exposure to anthrax would occur at one's home administrative unit--an assumption that limits the fidelity of the model.

**Methods:**

We employed commuting data provided by the U.S. Census Bureau to parameterize a commuting model. Then, we developed methods for integrating commuting into BARD's simulation and detection algorithms and conducted two studies to measure the effect. The first study (simulation study) was designed to assess how BARD's detection and characterization performance are impacted by incorporation of commuting in BARD's outbreak-simulation algorithm. The second study (detection study) was designed to measure the effect of incorporating commuting in BARD's outbreak-detection algorithm.

**Results:**

We found that failing to account for commuting in detection (when commuting is present in simulation) leads to a deterioration in BARD's detection and characterization performance that is both statistically and practically significant. We found that a simplified approach to accounting for commuting in detection--simplified to maintain tractability of inference--nearly fully restored both detection and characterization performance of BARD detector.

**Conclusion:**

We conclude that it is important to account for commuting (and mobility in general) in BARD's simulation algorithm. Further, the proposed method for incorporating commuting in BARD's detection algorithm can successfully perform the necessary correction in the detection algorithm, while preserving BARD's practicality. In our future work, we intend to further study the problem of the trade-off between running time and accuracy of the computation in BARD's version that includes commuting and ultimately find the best such trade-off.

## Introduction

Disease surveillance has a long history in Public Health. However, recent years have witnessed a sharp increase in the research devoted to the very early detection of disease outbreaks [1,2]. The diseases that have received special attention include those that occur naturally, especially the emerging infectious diseases, and those that may be caused by acts of bioterrorism. The detection of outbreaks is typically achieved through the analysis of various types of pre-diagnostic disease data, such as the counts of Emergency Department (ED) visits or the sales of over-the-counter healthcare products. To increase the utility of these data for detecting outbreaks of specific diseases, surveillance systems typically categorize the data according to various medical criteria. For example, the category of ED visits with influenza-like illness may be analyzed to detect influenza outbreaks. Similarly, the category of ED visits with respiratory chief complaints (RCC) may be analyzed to detect outbreaks of a respiratory disease, such as the disease caused by the inhalation of *B. Anthracis *spores. The use of such pre-diagnostic and categorized data for the purpose of detecting disease outbreaks is sometimes known as *syndromic surveillance*.

The early detection of outdoor aerosol releases of anthrax is an important problem because a release could infect hundreds of thousands of individuals and without early detection mortality could be as high as 30,000 to 3 million [3]. Further, the accurate characterization of an aerosol release in terms of time, location and quantity might assist responders in mitigating the impact of an outbreak. The Bayesian Aerosol Release Detector (BARD) is a system for detecting and characterizing releases of aerosolized anthrax. BARD integrates the analysis of biosurveillance data (counts of ED visits with RCC), meteorological data and geographical data. Through this analysis BARD attempts to determine whether the current spatio-temporal pattern of respiratory disease incidence in a region is more consistent with the historical patterns or with the pattern that BARD would expect with an aerosol anthrax release.

Modelling a population's exposure to aerosolized anthrax poses a number of challenges. A major difficulty is to accurately estimate the exposure level--the number of inhaled anthrax spores--of each individual in the exposed region. A key determinant of the exposure level of an individual is his/her spatial location at the time of exposure. Hence, to accurately estimate the exposure level it would be desirable to perform a fine-grained spatial modelling at the person-level. However, the data needed to parameterize such models are difficult to obtain. In fact, the only type of spatial information that is typically contained in biosurveillance databases is the home administrative unit--such as the home zip code--of each patient. To deal with this challenge posed by the lack of fine-grained spatial data, nearly all anthrax biosurveillance systems, including BARD, make two simplifying assumptions. First, they assume that all individuals who live in the same administrative unit would have the same exposure level--typically taken to be the level at the unit's centroid. Second, they assume that exposure to anthrax would occur at one's home administrative unit. Whether it is possible to relax the first assumption remains currently an open problem. On the other hand, in the last few years there has been some progress toward relaxing the second assumption. The key to this progress has been to integrate biosurveillance systems with mobility models that are parameterized through data that describe the travel patterns of the population. For one type of travel, namely the work-related commuting, such datasets are publicly available from the U.S. Census Bureau. For other types of travel, data are more difficult to obtain.

The first study that incorporated a mobility model in the simulation of anthrax outbreaks was conducted by Buckeridge [4]. He developed methods for integrating mobility in outbreak simulation and employed commuting data provided by the U.S. Census Bureau as well as survey based, non-commuting travel data. Then, he investigated the impact that incorporation of mobility in outbreak simulation had on two outbreak detection algorithms: a cumulative-sum temporal algorithm [1,2] and the SMART spatial algorithm [5]. A few other papers have investigated incorporation of mobility in biosurveillance. Duczmal and Buckeridge [6] proposed a method for integrating a commuting model with Kulldorf's spatial scan algorithm [7]. Garman et al. [8] developed a method for probabilistically inferring the work zip code from the home zip code and then integrated this method with the PANDA detection algorithm [9]. Cami et al. [10] developed a method for integrating commuting with BARD detection algorithm.

This paper describes a method for incorporating commuting into BARD. First, we incorporate commuting into BARD's outbreak-simulation algorithm. Then, we present the results of an experimental study that assessed how BARD's detection and characterization performance are impacted as a result. Next, we present the results of a second study designed to measure the effect of incorporating commuting in outbreak *detection*. Finally, we compare the results of the simulation and detection studies, discuss the findings and limitations of this work and outline some directions for future research.

## Background

### A brief description of BARD

Here we provide a brief description of BARD. A thorough discussion of this system can be found in Hogan et al. [3]. To simplify our exposition, we follow the terminology in Lawson and Kleinman [1] and generically refer to the basic administrative spatial units employed for aggregating the disease data (e.g., block groups or zip codes) as *tracts*. The main symbols used in this paper and their respective meanings are listed in Table 1.

**Table 1:** List of symbols

**Symbol**	**Meaning**
*t*	release date and time
*x*, *y*, *h*,	release coordinates
*q*	release quantity
*n*_*i*_	population of tract *i*
*c*_*i*_	number of cases who live in tract *i*
*d*_*i*_	dose of spores in the centroid of tract *i*
*θ*_*i*_	ED-visit probability of each individual who lives in tract *i*, given that a release has occurred and assuming that commuting has *not *taken place
*G*	commuting graph: nodes denote tracts, arcs denote commuting flows
*n*_*ij*_	for *i *≠ *j*, number of people who live in tract *i *and work in tract *j*; for *i *= *j*, number of people who live in tract *i *and who either don't work or work in tract *i*
*c*_*ij*_	for *i *≠ *j*, number of cases who live in tract *i *and work in tract *j*; for *i *= *j*, number of cases who live in tract *i *and who either don't work or work in tract *i*
*Out *(*i*)	union of set {*i*} with the set of all tracts where people who live in tract *i *work
	ED-visit probability of each individual who lives in tract *i*, given that a release has occurred and assuming that commuting has taken place

#### BARD detector

BARD detector attempts to discriminate between two hypotheses. The null hypothesis states that only "background" respiratory disease is present in the surveillance region. The alternative hypothesis states that both background respiratory disease and a respiratory disease caused by an aerosol release of anthrax are present. Using Bayes' theorem, BARD computes the posterior probability of the attack hypothesis *H*_1 _given data, as follows:

(1)

In addition, BARD computes the likelihood ratio, or the Bayes' factor

(2)

In equations (1)-(2), **b **is the *biosurveillance vector *consisting of the tract-level counts of ED visits with RCC during the last 24 hours; **G **is the *geographical matrix *containing the tract populations and the *x*, *y *coordinates of the tract centroids; **M **is the *meteorological matrix *containing the wind speed, the wind direction and the atmospheric stability class--a measure of atmospheric turbulence--for various observation times during the most recent week. The posterior probability of *H*_1 _and the Bayes' factor are both measures of the evidence provided by the data in favour of the alternative hypothesis.

The two key quantities in equations (1)-(2) are *P*(**b**|*H*_0_, **G**, **M**), the likelihood of biosurveillance data under *H*_0_, and *P*(**b**|*H*_1_, **G**, **M**), the likelihood of biosurveillance data under *H*_1_. Assuming conditional independence among the counts of different tracts given a hypothesis and the meteorological conditions, BARD computes the two region-wide likelihoods *P*(**b**|*H*_0_, **G**, **M**) and *P*(**b**|*H*_1_, **G**, **M**) by first computing the corresponding tract-level likelihoods and then using equations:

(3)

In eq. (3), *c*_*i *_denotes the 24-hr count of tract *i *and **r **=(*x*, *y*, *h*, *q*, *t*) denotes the release parameter vector, consisting of the release location *x*, *y*, *h*, the release quantity *q*, and the release time *t*. To compute the tract-level likelihoods *P(c*_*i *_|*H*_0_, **G**, **M**) and *P(c*_*i *_|*H*_1_, **G**, **M**, **r**), BARD employs the binomial probability model (see [3,10] for more details). Finally, BARD performs the integration over the release scenarios **r **through a Monte Carlo integration technique called *likelihood weighting *[3].

In addition to computing the two detection statistics discussed above--the posterior probability of *H*_1 _and the Bayes' factor--BARD attempts to characterize a release by computing an estimate for each element of the release vector **r**. The estimation of **r **is carried out in Bayesian fashion. BARD assumes that the release parameters are conditionally independent given *H*_1_, i.e.,

(4)

BARD employs a uniform prior for each element of **r**, except *h*, for which a prior that favours smaller release heights relative to the higher ones is employed (see [3], p. 5248). Finally, BARD computes the posterior expectation of each element of **r **inside the likelihood-weighting integration procedure. The posterior expectation of **r **constitutes the release characterization produced by BARD.

#### BARD simulator

BARD can also simulate outbreaks of the respiratory disease caused by an aerosol release of anthrax. Algorithm 1 gives a high-level description of BARD's outbreak simulation algorithm.

Algorithm 1: BARD outbreak simulation

**input**: surveillance region, release date/time, location, and quantity

**output**: list of simulated cases for each tract of surveillance region

**foreach **tract *i *of surveillance region **do**

1. compute the *dose of spores d*_*i *_in the centroid of tract *i *using the Gaussian plume model (see [3])

2. compute the probability *θ*_*i *_that an individual who lives in tract *i *will visit an ED, given the dose of spores *d*_*i *_(see [3])

3. generate the number of cases *c*_*i *_for tract *i *by drawing a random variate from *Bin(n*_*i*_, *θ*_*i*_), where *n*_*i *_is the population of tract *i*

4. **foreach **case of tract *i ***do**

      a. draw a random time interval from a *lognormal distribution *corresponding to the dose *d*_*i *_(see [3])

      b. add this interval to the release date/time to obtain the time of ED visit for this case

   **end**

end

For brevity, in Algorithm 1 we have left out a number of details that are not necessary for understanding the flow of the computation. These details can be found in [3].

## Methods

### Biosurveillance data

Our disease data--counts of ED visits with RCC--correspond to a region surrounding the city of Pittsburgh. This region consists of seven counties: Allegheny, Armstrong, Beaver, Butler, Lawrence, Washington, and Westmoreland. The historical ED data for our experiments were provided by 10 EDs operated by one health system. These data represented nearly 30% of all ED visits in the surveillance region. We divided the total period spanned by the available ED data into a training period (1 January 1999 to 31 December 2004), which was used to train BARD's detection algorithm, and a test period (1 January 2005 to 31 December 2005), used in our evaluation experiments. Finally, the weather data were provided from the National Weather Service, while the populations and central zip codes came from the ESRI ArcGIS Desktop product.

### Data and model for commuting flows

The data that describe the commuting patterns were provided by the U.S. Census Bureau. These data were collected during the 2000 Census, have national coverage and are provided at the *census tract *level: each commuting flow denotes the average daily number of commuters between a home census tract and a work census tract. The commuting flows can be naturally modelled by a weighted, directed graph in which nodes denote tracts, arcs represent commuting flows, and the weight of an arc denotes the number of commuters in the corresponding flow. We extracted from the nationwide commuting dataset the commuting flows for which both the residence tract and the work tract belong to our surveillance region. The total number of commuters in this intra-region subset of flows was 1,005,566.

As a pre-processing step, we needed to transform the commuting flows from the census tract level provided by the U. S. Census Bureau to one of the two levels supported by the BARD simulator: zip code, or block group. In this paper we performed the latter transformation mainly because the block-group level is finer-grained than the zip-code level and thus has the potential to lead to a more accurate estimation of the release location by BARD. Note that block group is a sub-unit of census tract (i.e., each census tract is partitioned into two or more block groups). To perform the desired transformation, we split each commuting flow between a pair of census tracts *T*_1_, *T*_2 _into several smaller-sized flows according to the following two intuitive rules: (i) the number of workers coming from each constituent block group of *T*_1 _was assumed to be proportional to the block group population; (ii) the number of workers going to *T*_2 _was assumed to be evenly divided among its constituent block groups. The final commuting graph for our region consisted of 1991 nodes (block groups) and 324,402 arcs (commuting flows).

### Integration of commuting with BARD simulator

We refer to the version of BARD simulator that takes commuting into account as the BARD-C simulator. BARD-C simulator takes as input a representation of the commuting graph *G*, in addition to the surveillance region and the anthrax release parameters. The key observation employed in the incorporation of commuting in simulation is that if a release occurs during working hours, a worker would be exposed at the dosage level corresponding to his/her work tract rather than the dosage level of his/her home tract. This idea is implemented in BARD-C by modifying the Step 3 of Algorithm 1, namely the computation of the ED-visit count for each tract. To realize this modification BARD-C first computes the ED-visit count corresponding to each commuting flow separately and then re-aggregates the workflow-level counts according to the home tract. More precisely, the number of cases for each tract *i *is computed as

(5)

where

(6)

In eqs. (5)-(6), *c*_*i *_denotes the total number of cases who live in tract *i*, *Out*(*i*) ={*i*} ∪ {*j*|(*i*, *j*) is an arc of *G*} denotes the union of set {*i*} with the set of out-neighbours of tract *i *in the commuting graph *G *(i.e., the tracts where people living in tract *i *go to work), *n*_*ij*_, *i *≠ *j*, denotes the weight of the arc (*i*, *j*) in the graph *G*, i.e., the number of people living in tract *i *and working in tract *j*, and *n*_*ii *_denotes number of people who either don't work, or who both live and work in tract *i*. Finally, *c*_*ij*_, *i *≠ *j*, denotes the number of cases who live in tract *i *and work in tract *j*, *c*_*ii *_denotes number of cases who either don't work, or who both live and work in tract *i *and *θ*_*j *_denotes the probability that a person *who is exposed in tract j *will visit an ED, given that a release has occurred.

This refined computation of the tract-level ED counts in BARD-C could alternatively be implemented by modifying the ED-visit probability of each individual who lives in tract *i *(i.e., modifying the Step 2 of Algorithm 1) prior to generating the number of cases for tract *i *as a binomial random variate. Indeed, let us denote by  the probability that an individual *who lives in tract i *will visit an ED, given that a release has occurred and assuming that commuting has taken place. Let the parameter *θ*_*i *_denote, as before, the probability that an individual *who is exposed to anthrax in tract i *will visit an ED. By approximating the binomial distribution with a Poisson distribution and recalling that the sum of independent Poisson random variables is also a Poisson random variable, it is straightforward to show that the method for generating the count *c*_*i *_specified by eqs. (5)-(6) is essentially the same as generating the count *c*_*i *_from a binomial distribution *Bin(n*_*i*_, ), where

(7)

The above two methods for incorporating commuting in simulation differ slightly from the method employed by Buckeridge [4]. In [4], other non-commuting types of mobility were also taken into account and therefore the exposure tract could be different from both the residential and the work tract. Hence, in [4] the incorporation of mobility was based on the computation of conditional probabilities P(exposed in tract *i*|*t*, live in tract *j*) for all pairs *i*, *j*.

After generating the number of cases for each tract, BARD-C computes the ED-visit time of each simulated case by, again, taking into account the fact that a worker would be exposed at the dosage level of his/her work tract. This idea is implemented in BARD-C by ensuring that in the Step 4(a) of Algorithm 1 the time of ED visit for a worker case is computed as a random deviate from the lognormal distribution that corresponds to the dosage level at his/her work tract.

The incorporation of commuting in simulation leads to an increase in the running time of the Step 3 of BARD's simulation algorithm by a constant factor. It is straightforward to see that this factor equals the average out-degree of the commuting graph. For the block group-level commuting graph of the Pittsburgh region that we created, the average out-degree is nearly 150. However, the factor by which the total running time of BARD simulator is increased due to the incorporation of commuting is smaller. The reason for this is that Step 3 accounts for only a fraction of the running time of BARD. In a computer with a 3 GHz processor and 2 GB of main memory, a simulation for the Pittsburgh region took nearly 2 minutes with BARD and nearly 20 minutes with BARD-C.

### Verification of the correctness of simulation with commuting

To verify that BARD-C simulator works as intended, we generated a random release parameter vector, consisting of the release time, location, and quantity. Then, using this fixed release parameter vector we produced 10 synthetic outbreaks with BARD-C simulator. Next, for each tract *i *of the surveillance region, we fit a binomial model *Bin*(*n*_*i*_, ) to the sample of simulated counts . As before, *n*_*i *_denotes the known population of tract *i*, and  denotes the probability that an individual *who lives in tract i *will visit an ED, given that a release has occurred and assuming that commuting has taken place. We denote by  the estimate of the binomial probability parameter  learned from the count data generated with BARD-C simulator. Finally, we computed an analytical prediction for each parameter . We achieved this by first using the BARD simulator to compute the binomial probability parameters *θ*_*j *_for all *j *∈ *Out*(*i*) and then computing the analytical prediction of the parameter  through eq. (7).

### Integration of commuting model with BARD detector

We have also developed a method for integrating commuting with BARD detector. A thorough discussion of this method is given [10]. We refer to the version of BARD detector that takes commuting into account as the BARD-C detector.

### Measuring the impact of commuting in BARD's performance: simulation study

**Figure 1:**
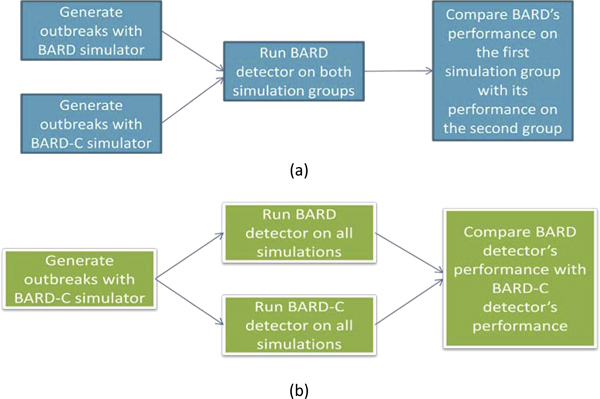
**Schematic representation of the simulation and detection studies**. Part (a): simulation study; part (b): detection study

We conducted an experiment to measure whether BARD's detection and characterization performance is significantly affected by the incorporation of commuting in outbreak simulation. In the remainder of the paper, we refer to this experiment as the "simulation study" because the emphasis of this study was on the integration of commuting with BARD simulator. In the next section we discuss a second study, where the emphasis is on the integration of commuting with BARD detector. We refer to this second study as the "detection study". Figure 1 illustrates the difference between the objectives of the simulation and detection studies.

The simulation study followed a matched-pairs design. First, a set of 185 release parameter vectors **r **= (*t*, *x*, *y*, *h*, *q*) was generated. For all parameter vectors, the quantity *q *was fixed at 0.5 kg. The release time *t *was chosen uniformly at random from the test period, i.e., the year 2005. The parameters *x *and *y *where chosen uniformly at random from a circular sub-region of the surveillance region centred in the city of Pittsburgh and having a radius of 40 km; this sub-region was found to have better ED-data coverage than the whole region. Finally, the height *h *was chosen from a non-uniform distribution that favours lower release heights relative to the higher ones.

We supplied each release vector **r**_*i*_, *i *= 1,...,185, as input to the BARD and BARD-C simulators. By doing so, we obtained one group of 185 simulations that did not take commuting into account and another group of 185 simulations that took commuting into account. We refer to these two simulation groups as *non-commuting *and *commuting*, respectively. The synthetic cases generated in each simulation were injected into the real ED data from 2005. Next, each of the 370 semi-synthetic outbreaks was supplied as input to the BARD detector and its average performance over the non-commuting outbreak group was compared with its average performance over the commuting outbreak group.

BARD's detection performance was measured through the time-to-detection--or *timeliness--*and its characterization performance was measured through the *t, x, y, h*, and *q *(*absolute*) *errors*. Each of the above six metrics is a function of the *alarm threshold*, i.e., the threshold of the alarm statistic employed by BARD detector to discriminate between the non-outbreak and outbreak situations. For a given threshold, an alarm is considered to be false if the alarm statistic exceeds the threshold prior to the outbreak onset. The *false alarm rate *(for a given alarm threshold) is the number of false alarms that occur per unit of time. We measured it as the number of false alarms per year.

We employed two methods to compare BARD's performance over the non-commuting group with its performance over the commuting group. The first method was Activity Monitoring Operating Characteristic (AMOC) analysis [11]. The AMOC curve of a performance metric (e.g., timeliness) plots the false alarm rate against that metric. The initial step in constructing an AMOC curve is to compute the alarm thresholds corresponding to various false alarm rates (in our case, 1 per year to 36 per year). We computed the alarm thresholds by running BARD detector on the baseline ED data for 2005. Next, these thresholds were employed to identify the pairs of values of the form <*false alarm rate, performance metric*> that constitute an AMOC curve. For each simulation group, we constructed a single AMOC curve per metric by plotting the false alarm rate against the *mean *value of the given metric over the whole simulation group.

The second method we employed to compare BARD's performance over the non-commuting group with its performance over the commuting group was statistical testing. Our null hypothesis asserted that for a given false alarm rate and performance metric, the mean of the given metric over the non-commuting group was equal to its mean over the commuting group. Testing was carried out through the *paired t test*.

### Comparing the performance of BARD and BARD-C detectors: detection study

We performed a second experiment designed to compare the performance of BARD and BARD-C *detectors *over a set of simulations generated with BARD-C simulator. As mentioned earlier, we refer to this experiment as the "detection study". Here, we briefly outline the design of this study; a full discussion is given in [10]. A total of 100 semi-synthetic outbreaks were generated using the BARD-C simulator. For all simulations, the quantity *q *was fixed at 0.5 kg. The release time *t *was chosen uniformly at random from the test period, i.e., the year 2005. The parameters *x*, *y*, and *h *were drawn from their prior distributions. Each semi-synthetic outbreak was supplied as input to the BARD and BARD-C detectors. The detectors were executed 42 times on each simulation in increments of 4 hours: the first execution began 4 hours after the release, the second execution 8 hours after the release, and so on.

The *detection performance *of both detectors was measured through *timeliness*. AMOC analysis and statistical testing were employed to compare the performance of the two detectors. The *characterization performance *of both detectors was measured through *t*, *x*, *y*, *h*, and *q *(*absolute*) *errors*. Each characterization metric was plotted against the time interval from the release of anthrax to the beginning of the detector's execution, which we call the *time-to-execution*. Finally, a Wilcoxon signed ranked test was performed with respect to each characterization error.

## Results

### Correctness of simulation with commuting

**Figure 2:**
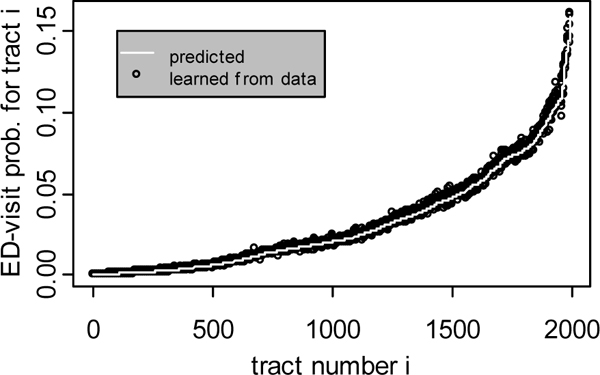
**Verification of the correctness of simulation with commuting**. Plot of the analytically predicted values of ED-visit probability against the values estimated from data generated with the BARD-C simulator

Figure 2 shows the results of our experimental verification of the correctness of simulation with commuting. This figure plots the estimated parameter values  and the analytically predicted values  for the 1991 tracts of our surveillance region. The solid line corresponds to the predicted values, while the points correspond to the values estimated from the simulated data. As shown, the predicted values are in good agreement with the values estimated from the simulated data, indicating that BARD-C simulator works as specified.

### Results of the simulation study

**Figure 3:**
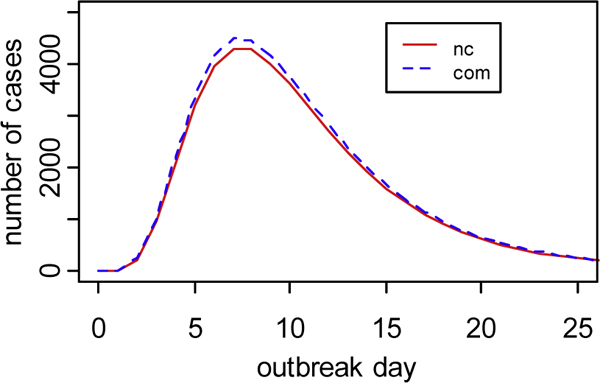
**ED visit counts generated with BARD and BARD-C simulators**. Daily mean number of ED visits in the surveillance region for the non-commuting (nc) and commuting (com) simulation groups

Figure 3 shows the daily mean number of cases (i.e., the epidemic curves) for the non-commuting and commuting simulation groups. In this figure, the horizontal axis shows the outbreak day, while the vertical axis shows the daily mean number of cases in the surveillance region. It can be observed that the shapes of these two curves are very similar. In particular, the two curves are seemingly identical at least up to the third outbreak day--the day when BARD typically detects a semi-synthetic outbreak. These two curves differ mainly around the peak of the outbreak, where the commuting curve is slightly more elevated than the non-commuting curve. These observations imply that any differences between BARD detector's performance over one simulation group and its performance over the second group could be attributed only to the change in the spatial distribution of cases caused by commuting and not to a consistent discrepancy between the sizes of outbreaks simulated with and without commuting.

With respect to *q *and *h *errors, we found that BARD's performance over the non-commuting group was almost indistinguishable from its performance over the commuting group. Unexpectedly, the posterior mean of the release parameters *q *and *h *did not appear to converge. We leave the investigation of this unexpected outcome as a topic for future research and in the remainder of this section we focus only on the remaining four metrics.

**Figure 4:**
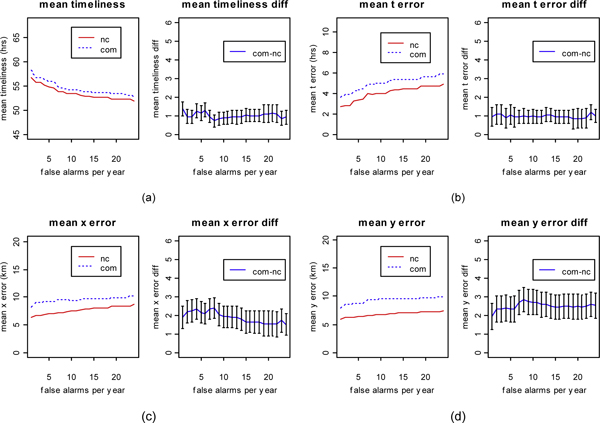
**AMOC analysis for the simulation study**. AMOC curves for (a) mean timeliness, (b) mean t error, (c) mean x error, and (d) mean y error. In each of the parts (a)-(d), the left panel shows the AMOC curves corresponding to the commuting (com) and non-commuting (nc) simulation groups, respectively, while the right panel shows the difference between commuting and non-commuting AMOC curves along with the error bars (plus/minus one standard error) for this difference

Figure 4 shows the results of the AMOC analysis for the timeliness, *t *error, *x *error, and *y *error. As shown, in Figure 4(a), BARD's mean timeliness over the commuting group is nearly one hour greater than its mean timeliness over the non-commuting group, at each false alarm rate. Likewise, BARD's mean *t *error (Figure 4(b)) over the commuting group is nearly one hour greater than its mean *t *error over the non-commuting group, at each false alarm rate. BARD's mean *x *and *y *errors (Figure 4(c-d)) on the commuting group are each nearly 2 km greater than the respective mean errors on the non-commuting group.

Finally, Table 2 gives for all false alarm rates between 1 per year and 12 per year the p-values of the test for equal means of BARD's timeliness, *x *error, *y *error, and *h *error over the two simulation groups. From the results shown in Table 2 it can be concluded that, at each shown false alarm rate, the mean timeliness, *x *error, and *y *error over the non-commuting group are statistically different from the respective means over the commuting group, at the 0.05 significance level. The same holds for the *t *error with the exception of three false alarm rates: 1, 4, and 6.

**Table 2:** Results of the test for equality of BARD's errors over the two simulation groups.

	**timeliness**	**t error**	**x error**	**y error**
**far = 1**	0.00014	0.06698	0.00139	0.0055
**far = 2**	0.00889	0.04066	0.00026	0.00106
**far = 3**	0.00991	0.03827	0.00024	0.00107
**far = 4**	0.00074	0.09732	0.00006	0.00061
**far = 5**	0.0046	0.04967	0.00017	0.00086
**far = 6**	0.00044	0.07566	0.0002	0.00068
**far = 7**	0.0041	0.01017	0.00004	0.00008
**far = 8**	0.03221	0.01087	0.00003	0.00003
**far = 9**	0.01664	0.00879	0.00059	0.00006
**far = 10**	0.01007	0.00689	0.00104	0.00006
**far = 11**	0.00819	0.00762	0.00107	0.00007
**far = 12**	0.00477	0.00775	0.00124	0.00013

The p-values of the test H_0_: mean(metric) non-commuting equals mean(metric) commuting--where metric can be timeliness, t error, x error, or y error.

### Results of the detection study

**Figure 5:**
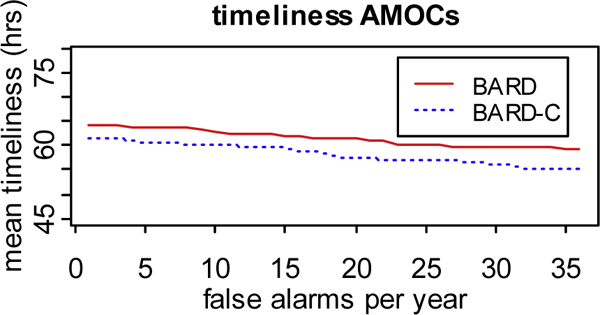
**AMOC analysis for the detection study**. AMOC curves for the mean timeliness of BARD and BARD-C detectors measured over a group of simulations generated with BARD-C simulator

**Figure 6:**
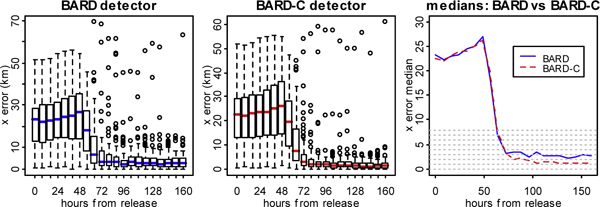
**Analysis of the characterization performance for the x error**. Box plots and plot of the medians of the *x *error for BARD and BARD-C detectors

**Figure 7:**
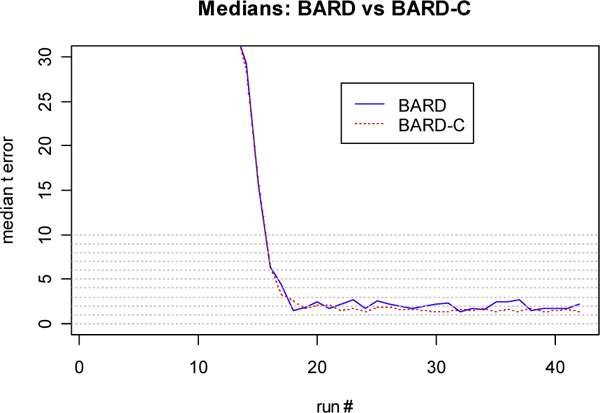
**Analysis of the characterization performance for the y error**. Plot of the median of the *y *error for BARD and BARD-C detectors

**Figure 8:**
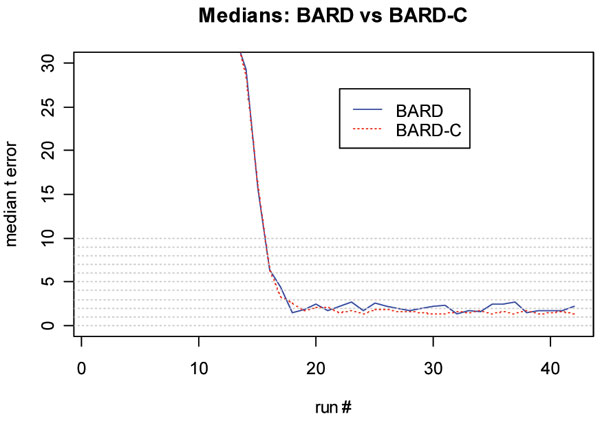
**Analysis of characterization performance for the t error**. Plot of the median of the *t *error for BARD and BARD-C detectors

Some of the results appearing in this section (Figs 5 and 6) have been previously reported in [10]. We have reproduced these results here to contrast the simulation study with the detection study. This section also presents two new plots (Figs 7, 8) that correspond to our detection study but do not appear in [10]. Figure 5 shows the AMOC analysis for the timeliness of BARD and BARD-C detectors. As seen, BARD-C's timeliness is more than one hour smaller than BARD's timeliness at every false alarm rate. A paired *t *test showed that, for each false alarm rate, the mean timeliness of BARD-C was statistically different from the mean timeliness of BARD, at the 0.05 significance level.

Next, we turn to the characterization performance of BARD and BARD-C detectors. As in the simulation study, we found that the posterior means of the release parameters *q *and *h *did not appear to converge as the time to execution increased.

Hence, in the remainder of this section we focus on the *x*, *y*, and *t *errors. Figure 6 shows box plots and plots of the medians of the *x *error for BARD and BARD-C. In each plot the horizontal axis shows the time-to-execution. The samples from which the box plots were constructed correspond to the different simulations. Several conclusions can be derived from Figure 6. First, the *x *error of both BARD and BARD-C converges to relatively small values as the time-to-execution increases. Second, the errors of BARD and BARD-C appear to converge around the same time and nearly 10-16 hours after the detection. Third, the sampling distribution of the *x *error is right-skewed for both detectors and in almost each execution of BARD and BARD-C there is a small number of outliers. Fourth, after the convergence, the median of the *x *error for BARD-C is nearly 2 km smaller than the median of the *x *error for BARD.

Figure 7 plots the median of the *y *error for BARD and BARD-C. The post-convergence median of the *y *error for BARD-C was nearly 0.5 km smaller than the median for BARD. Finally, Figure 8 plots the median of the *t *error for BARD and BARD-C. The post-convergence difference of the medians for the *t *error was nearly 20 minutes.

Finally, we performed a Wilcoxon signed rank test whose null hypothesis asserted that BARD and BARD-C have identical locations of the distributions of *x *error, *y *error, and *t *error. The results of this test showed that after the convergence of the *x *error, the p-values of the test remain well below the 0.05 significance level (see [10]). Similar comments can be made for the *y *error. For the *t *error the convergence of the p values is not as clear as for the *x *and *y *errors.

## Conclusion

We incorporated commuting into BARD's simulation and detection algorithms and conducted two experimental studies to evaluate the effect. Incorporation of commuting in simulation is important to improve the fidelity of semi-synthetic outbreaks that BARD generates. Likewise, by incorporating commuting in detection we expect that the laboratory performance of the BARD detector would be closer to a real-world performance.

The results of our simulation study showed that incorporation of commuting in outbreak simulation leads to a statistically significant deterioration in BARD's detection and characterization performance, when BARD's detection algorithm does not account for commuting. Intuitively, this result was expected since incorporation of commuting in simulation causes the spatio-temporal pattern of respiratory disease incidence to be different from the pattern BARD would expect under the alternative hypothesis. However, our precise quantification of the deterioration in BARD's performance due to commuting showed that this deterioration is practically significant. Indeed, BARD's timeliness increased by nearly one hour and based on previous estimates [12] that a delay of one hour in detection results in as much as $250 million additional economic costs, this increase is quite significant. Likewise, the *t, x, y *errors increased by nearly 30%, which can also be considered a significant deterioration.

Our detection study showed that incorporation of commuting in detection almost fully restores BARD's performance in the sense that the performance of BARD-C detector on simulations produced with the BARD-C simulator is close to the performance of BARD detector on simulations produced with BARD simulator.

Although others have measured the effect of population mobility on outbreak-detection performance, to our knowledge our work is the first to report the effect on outbreak-characterization performance. We found that failing to account for commuting in detection (when commuting is present in simulation) worsens characterization performance. We found that our simplified approach to accounting for commuting in detection--simplified to maintain tractability of inference--significantly improved characterization performance over characterization without commuting.

Finally, we discuss the main limitations of our work. First, in creating the commuting graph of the surveillance region, we employed intuitive but arbitrary rules to transform the commuting flows into a spatial resolution supported by BARD. The need for transforming the commuting flows could be avoided in the future by extending BARD so that it supports the census tract mode, but at the expense of coarser spatial granularity. Second, although we did not use the same commuting graph in both simulation and detection (see [10] for the details of how we truncated and sorted the commuting graph in detection mode) our studies did not perform a full sensitivity analysis with respect to the commuting graph. In our future work we plan to create a number of commuting graphs, each obtained by introducing random noise to the commuting data provided by the Census Bureau. Then, we will simulate several synthetic outbreaks with each of these noisy graphs and will compare BARD and BARD-C detectors over the whole range of simulations. Finally, as elaborated in [10], to bound the running time of the BARD-C detector, we made a number of simplifications at the expense of the accuracy of the computation. In our future work, we intend to further study the problem of the trade-off between running time and the accuracy of the computation in BARD-C detector and ultimately find the best such trade-off.

## Competing interests

The authors declare that they have no competing interests.

## Authors' contributions

AC, GLW and WRH developed the proposed methods, designed the experimental studies and prepared the manuscript. AC implemented the proposed methods and performed the experiments.
